# Supplementary material for: The sperm hook as a functional adaptation for migration and self-organized behavior
Source: eLife. 2024 Nov 22;13:RP96582. doi: 10.7554/eLife.96582 (PMC11584178; doi:10.7554/eLife.96582)
Supplement: Supplementary file 1. — (a) Basic information of 4 males that were used for the mating experiment. (b) Mating records and the information of the females for sperm tracking. (c) Summary results of the GLMM. [file elife-96582-supp1.docx]

Supplementary File 1a. Basic information of 4 males that were used for the mating experiment.

| Male ID | Date of Birth (DOB) | Genotype | Note |
| --- | --- | --- | --- |
| A (No. 838) | 2021.04.21 | RBGS-CX3CR1 |  |
| B (No. 3399) | 2021.08.17 | RBGS |  |
| C (No. 3613) | 2021.09.01 | RBGS |  |
| D (No. 3610) | 2021.09.01 | RBGS-CX3CR1 | Vasectomized male |

RBGS: CAG/Su9-DsRed2 and Acr3-EGFP, RBGS-CX3CR1: CAG/Su9-DsRed2, Acr3-EGFP, and Cx3cr1

Supplementary File 1b. Mating records and the information of the females for sperm tracking.

| Date of mating | DOB (female) | Age (female) | Male ID |
| --- | --- | --- | --- |
| 2021-12-15 | 2021-09-01 | 15.0 weeks | A |
| 2021-12-23 | 2021-07-02 | 24.9 weeks | B |
| 2022-03-11 | 2021-05-10 | 43.6 weeks | A |
| 2022-04-07 | 2021-08-17 | 33.3 weeks | A |
| 2022-06-08 | 2021-10-17 | 33.4 weeks | C |
| 2022-07-07 | 2022-02-14 | 20.4 weeks | C |
| 2022-07-14 | 2021-10-17 | 38.6 weeks | A |
| 2022-08-04 | 2022-02-14 | 24.4 weeks | A |

Male ID: males (Supplementary Table 1) who mated with the subject females on the date.

Supplementary File 1c. Summary results of the generalized linear mixed models (GLMM).

Each model represents sperm trajectory parameters that were log-transformed. In all models, we examined the effect of sperm to uterine wall distance (Distance from wall), angle between a sperm trajectory and uterine wall (respective angle with wall) and cropping of the acquired image (O: cropped vs X: uncropped). The GLMM for SWR showed a boundary (singular) fit warning message. However, two models that omitted one of random variables (Male or Date) did not result in any significant changes in the predictor variables (p > 0.05).

| Model | Predictors | Estimates | SE | *t* | *P* | 95% CI |
| --- | --- | --- | --- | --- | --- | --- |
| VCL | (Intercept) | 4.0645 | 0.4189 | 9.7028 | 0.001** | 3.198, 4.921 |
|  | Distance from wall | -0.0094 | 0.0007 | -12.8449 | <0.001*** | -0.011, -0.008 |
|  | Angle with wall | -0.0019 | 0.0007 | -2.6255 | 0.009** | -0.003, 0 |
|  | Cropped (O: X) | -0.2576 | 0.4368 | -0.5897 | 0.584 | -1.155, 0.652 |
| VSL | (Intercept) | 3.2745 | 0.4277 | 7.6558 | 0.002** | 2.434, 4.113 |
|  | Distance from wall | -0.0104 | 0.0014 | -7.4718 | <0.001*** | -0.013, -0.008 |
|  | Angle with wall | -0.0081 | 0.0014 | -5.9303 | <0.001*** | -0.011, -0.006 |
|  | Cropped (O: X) | -0.0822 | 0.4202 | -0.1957 | 0.853 | -0.902, 0.716 |
| LIN | (Intercept) | -0.8236 | 0.1194 | -6.8999 | 0.001** | -1.058, -0.581 |
|  | Distance from wall | -0.0009 | 0.0011 | -0.8632 | 0.388 | -0.003, 0.001 |
|  | Angle with wall | -0.0061 | 0.0010 | -5.8469 | <0.001*** | -0.008, -0.004 |
|  | Cropped (O: X) | 0.1701 | 0.1365 | 1.2459 | 0.273 | -0.113, 0.444 |
| SWR | (Intercept) | 1.1507 | 0.1458 | 7.8917 | <0.001*** | 0.732, 1.430 |
|  | Distance from wall | -0.0003 | 0.0013 | -0.2149 | 0.830 | -0.003, 0.002 |
|  | Angle with wall | -0.0108 | 0.0013 | -8.2828 | <0.001*** | -0.013, -0.008 |
|  | Cropped (O: X) | 0.1566 | 0.1720 | 0.9107 | 0.399 | -0.384, 0.835 |

VCL: curvilinear velocity, VSL: straight-line velocity, LIN: linearity of forward progression, SWR: straight line-to-sideward movement ratio, ***: p < 0.001, **: p < 0.01, *: p < 0.05.
